# Psychological issues in cleft lip and cleft palate

**DOI:** 10.4103/0971-9261.55152

**Published:** 2009

**Authors:** Avinash De Sousa, Shibani Devare, Jyoti Ghanshani

**Affiliations:** Consultant Psychiatrist, Mumbai, India; 1 Consultant Clinical Psychologists, Mumbai, India

**Keywords:** Cleft lip, cleft palate, psychological issues

## Abstract

Vocational and social issues affect rehabilitation and development of patients with cleft lip and cleft palate. However, psychological problems like lowered self esteem and difficulties in social interaction have also been noted in them. Not many pediatric reconstructive surgery teams have a psychiatrist on their panel. It is likely that psychological problems are higher in incidence than literature actually suggests. Hence it is very essential that such cases are identified by the surgical team to maximize positive outcome of surgery and rehabilitation. This study discusses psychological issues revolving around cleft lip and cleft palate along with lacunae in many psychological research studies.

## INTRODUCTION

Research shows, learning to live with a change in appearance of one's face as a result of injury or disease is a difficult task.[1] It is additionally challenging for children with congenital craniofacial conditions and their parents to adjust. Evidence shows, amongst the various craniofacial developmental abnormalities, cleft lip and cleft palate occur most commonly, affecting one in 700 live births.[2] The role of physical appearance has proved that a healthy physical appearance, regardless of facial or physical characteristics, is considered attractive.[3] Significant literature has shown, in addition to coping with their physical appearance, children with cleft anomaly in general have to deal with their more superficial psychological issues/ psychosocial limitations. Existing multispecialty care is primarily aimed at physical rehabilitation with the psychological issues of care often being neglected.[4] This paper discusses the various psychosocial issues amongst children and adults with cleft lip and cleft palate.

## OVERVIEW OF PSYCHOSOCIAL ISSUES IN CLEFT LIP AND CLEFT PALATE

Various physiological and sociocultural factors contribute in the development of psychosocial issues among individuals with any form of facial anomaly in general. Research has shown that attractive children are seen by others as brighter, having more positive social behavior and receive more positive treatment than their less attractive counterparts.[4,5] Self perception plays a pivotal role in influencing an individual's self esteem and psychological adjustment affected by cleft lip and palate anomaly.[6–8] Additionally, parental influence also shapes ones psychosocial perception. The attitudes, expectations and degree of support shown by parents can influence a child's perception of their cleft impairment.[9,10] Parents of children with clefts may be more tolerant of misbehavior in their child and are more likely to spoil their child by being overprotective.[4,11] Additionally, peer interaction also plays an important role in maintaining psychosocial limitations. Many children with cleft lip and palate may have a less attractive facial appearance or speech than their peers. A high incidence of teasing over facial appearance is reported among those with cleft lip and palate.[12–17] A self report research study on determining the psychosocial functioning related to cleft lip and palate, showed participants with cleft lip and palate reported greater behavioral problems; were teased often and less happy with their facial appearances.[12]

The study concludes that having been teased was a significant predictor of poor psychological functioning amongst individuals with cleft lip and palate. However, it found limited evidence to suggest that individuals may encounter psychosocial problems as a result of having a cleft lip and palate, with overall adjustment and functioning appearing to be reasonably good.

## SOCIAL STIGMA AND CLEFT DISORDERS

A social stigma is created within an individual when he/she is negatively discriminated by labeling him/her different from normal. An individual's thoughts, feelings and behavior related to their physical appearance makes their body image attitudes.[17] A negative response from outsiders, actual or perceived, may adversely affect self-image.[4,18,19] Also physical attractiveness plays an important role in the development and maintenance of self beliefs. Research indicates that preference for attractive individuals subsequently influences self esteem, social competence, and future ratings of attractiveness.[3] Moreover, being physically attractive appears to be an advantageous trait regardless of age. Physical attractiveness has shown to play a significant role in social set ups like developing relationships during various stages of life, school, courtships, work etc. Social acceptance often depends on one's physical look. These associations between physical beauty and social acceptability indicate the difficulties for cleft lip and palate affected individuals.[4,9]

## EDUCATION AND COMMUNICATION PROBLEMS

Evidence shows that communication problems related to cleft lip and palate are noticeable at a young age. A research study on the development of children with cleft lip and palate infants and toddlers, from birth till the age of three, reveals that toddlers with cleft palate exhibit ‘at-risk/delayed’ development in the expressive language domain at 36 months.[20] It is also observed that factors directly affecting the psychological development of a child born with cleft lip and palate include possible speech and language disorders, facial disfigurement, and hearing loss.[21] Research thus makes the association of communication problems with cleft lip and palate evident. To add on to the communication disadvantages experienced by individuals with cleft lip and palate, it becomes more difficult to deal with emotional issues during their academic years. Studies on cleft have shown relationships between (a) facial appearance and teacher perception, (b) behavioral inhibition and lower school achievement, and (c) speech defectiveness and self-esteem.[22]

Research shows that a high percentage of cleft children are underachievers along with the evidence of behavioral inhibition, concern regarding appearance, and decreased expectations by teachers and parents.

## PSYCHOLOGICAL FACTORS IN CLEFT SURGERY

It is evident that with various limitations that individuals with cleft lip and palate experience, they are bound to encounter various psychological difficulties. Moreover, these limitations build up over a period of time because of the psychological problems faced. For example, communication disorders in individuals with cleft lip and palate seem not to result from phonological defects but from psychological problems that may influence the entire development of an affected child.[23] Anxiety and depression have also been reported to be twice as prevalent in adults with cleft lip and palate compared with normal controls.[24] Difficulties are also experienced in relation to behavioral problems and satisfaction with facial appearances.[12] Moreover, these psychological problems can be interrelated. Anxiety, depression, and palpitations were reported about twice as often by subjects with cleft lip and palate compared with controls, and these psychological problems were strongly associated with concerns about appearance, dentition, speech, and desire for further treatment.[24] Additionally, findings in studies indicated that psychological and behavioral problems depended on the type of cleft deformity. For example, children with only cleft palate only showed greater problems with parents, reported depression, anxiety, and learning related to speech than children with unilateral cleft lip and palate or bilateral cleft lip and palate. The latter two groups showed fewer problems and a greater relationship of problem to facial appearance.[25] These psychological difficulties are not just limited to individuals/children with cleft lip abnormality, but also to their parents. Research studies have shown parents to experience mental crisis, based on their own previous background, coping with present stress etc. in bringing up a child with cleft lip.[4,26]

## SATISFACTION AND ISSUES AFTER SURGERY

Surgery, being the immediate option of dealing with certain issues related to disfigurement, is beneficial in dealing with both physical and psychological issues. Surgery usually results in increased self esteem, self confidence and satisfaction with appearance.[27] It can be used in young patients to improve esthetic appearance, an important factor in the psychological development of adolescents.[28] However, it is necessary for oneself to develop positive self skills to deal with the post surgery situations. Any individual with facial differences, who has fostered these skills, can achieve acceptance, develop positive social interaction skills, demonstrate social competence, and be less likely to exhibit significant adjustment problems.[29] Unrealistic, high expectations post surgery may also lead to dissatisfaction, which may further affect an individuals self satisfaction.[4] This disappointment and dissatisfaction can also be experienced by parents. Evidence makes the need for disseminating valuable information on the pros and cons of surgery essential. A study on patient satisfaction observed that majority of the patients expressed satisfaction on the care provided, 30% of the parents expressed a need to make them more involved treatment planning decision with most of them having no or inadequate knowledge on cleft lip treatment procedures.[30]

## PROBLEMS WITH CLEFT LIP AND PALATE PSYCHOLOGICAL RESEARCH

Though several research studies have been carried out on various aspects of cleft lip and palate they are insufficient in providing information. Studies do predict some amount of difficulties in psychosocial functioning among cleft lip palate individuals, however there is limited information on the severity and the duration of the same.[12] There is no direct evidence of cleft lip palate on behavior. Many studies have shown other environmental, confounding factors such as teasing, leading to poor psychological functioning, more so than having a cleft lip and/or palate per se,[21] thus providing conflicting evidence when it comes to establishing whether children and adults with cleft lip and palate experience psychological problems as a result of their cleft.

Many studies investigating psychological issues of cleft lip and palate use self reported data,[4,12] thus indicating a possible error of predisposition to self perception Similar self reports by parents and individual with cleft lip and palate have been reviewed to identify the level of satisfaction post surgery.[31] As observed, unrealistic expectations can also play a pivotal role in developing psychological distress. Research on determining the needs of parents as well as patients on cleft lip and palate would be useful in providing basic information of cleft lip palate and its characteristics pre and post surgery.

Additionally, facial growth would change along with age and treatment interventions; longitudinal studies which determine the facial growth changes and the experience of surgery should be examined as possible factors influencing psychosocial functioning.[4] An individual's personality traits i.e. level of confidence and environmental factors like upbringing, family background play a central role in influencing behavior. It appears that research studies of the effects of cleft related conditions on behavior should examine both internalizing and externalizing dimensions of behavior and consider that there may be subgroups of children showing these different kinds of behavior across different age levels.[21]

## CONCLUSION

Identifying the common psychosocial factors related to cleft lip and palate remains a major challenge. Extensive research data does suggest that psychological and psychosocial factors have an effect on behavior, but there is limited evidence to suggest that individuals experience psychosocial problems as a result of cleft lip and palate. More research is required to develop a tool whereby bias in self reporting could be avoided. Additionally, there is a need to evaluate patient and family before surgery and help provide them with relevant information on post and pre surgery issues.
